# A simple angle-measuring instrument for measuring cemented stem anteversion during total hip arthroplasty

**DOI:** 10.1186/s12891-020-3142-7

**Published:** 2020-02-19

**Authors:** Ryo Mitsutake, Hiromasa Tanino, Yasuhiro Nishida, Masaru Higa, Hiroshi Ito

**Affiliations:** 1 Department of Orthopaedic Surgery, Asahikawa Medical University, Midorigaoka-Higashi 2-1-1-1, Asahikawa, 078-8510 Japan; 2 Department of Mechanical Engineering, University of Hyogo, 2167 Shosha, Himeji, 671-2280 Japan

**Keywords:** Total hip arthroplasty, Stem anteversion, Angle-measuring instrument, Cemented stem

## Abstract

**Background:**

During total hip arthroplasty (THA), the accurate placement of the femoral components is an important determinant of the success of the procedure. This study assessed the accuracy of cemented stem placement using a new angle-measuring instrument. The primary objective was to investigate the accuracy of the intraoperative measurements of cemented stem anteversion obtained using the angle-measuring instrument. Our secondary objective was to evaluate the accuracy of stem positioning performed using the angle-measuring instrument.

**Methods:**

We compared the intraoperative stem anteversion measurements obtained using the angle-measuring instrument with postoperative stem anteversion measurements obtained using computed tomography in 149 hips (measurement accuracy). We also compared the target angle and postoperative stem anteversion in 105 hips (implantation accuracy).

**Results:**

The mean amount of intraoperative stem anteversion was 37.9° ± 10.1°, and the mean amount of postoperative stem anteversion was 37.0° ± 10.4°. The mean measurement accuracy was 0.9° ± 6.1°, and the absolute measurement accuracy was 4.9° ± 3.7°. The correlation coefficient for the relationship between the intraoperative and postoperative stem anteversion measurements was 0.824 (*p* = 0.000). The mean amount of target angle was 37.4° ± 7.6°, and the mean amount of postoperative stem anteversion was 35.9° ± 9.1°. The mean implantation accuracy was 1.4° ± 5.6°, and the mean absolute implantation accuracy was 4.3° ± 3.6°. The correlation coefficient for the relationship between the target angle and postoperative stem anteversion was 0.795 (*p* = 0.000).

**Conclusions:**

The angle-measuring instrument measured intraoperative stem anteversion accurately, and cemented stem was implanted accurately during THA with the angle-measuring instrument.

## Background

During total hip arthroplasty (THA), the accurate placement of the femoral and acetabular components is an important determinant of the early and long-term success of the procedure. Poor component orientation is associated with impingement, a reduced range of motion, dislocation, and increased component wear [1–7]. Many studies have reported the optimal positions for the acetabular components, and the accuracy of acetabular component positioning using free-hand techniques, mechanically guided techniques, or navigation systems have also been investigated [8–11]. Other studies have examined the accuracy of intraoperative measurements of stem anteversion obtained using a goniometer or navigation systems during THA [12–16]. However, only one CT-based navigation study has reported the accuracy of stem positioning during THA [17]. Moreover, no studies have reported the accuracy of stem positioning using a goniometer or mechanical guides. With cemented arthroplasty components, stem anteversion can be easily controlled by the surgeon. In contrast, in cementless arthroplasty, stem anteversion is determined by the anatomy of the femoral neck, the diaphyseal bow, and the anterior-posterior isthmus at the level of the lesser trochanter created by the calcar femorale [18]. To measure cemented stem anteversion intraoperatively during THA, we developed a simple angle-measuring instrument based on a goniometer. The primary objective of this study was to investigate the accuracy of intraoperative measurements of cemented stem anteversion obtained using the angle-measuring instrument. Our secondary objective was to evaluate the accuracy of stem positioning performed using the angle-measuring instrument.

## Methods

### Study population

The study protocol was approved by our hospital’s institutional review board (AMU 18183). Between August 2017 and November 2018, two experienced surgeons performed primary THA using a particular cemented femoral component in 142 patients (149 hips) at our hospital. During the same period, cementless THA was performed in patients aged < 50 years who had good-quality femoral cortical bone. *Measurement accuracy* was defined as the difference between the intraoperative measurements of stem anteversion obtained using the angle-measuring instrument and the postoperative measurements of stem anteversion obtained using postoperative computed tomography (CT). The measurement accuracy of the angle-measuring instrument during the measurement of cemented stem anteversion was investigated in 149 consecutive hips (Table 1).

**Table 1:** Subjects’ demographic data

Parameters	Measurement accuracy	Implantation accuracy
No. of hips/patients	149 hips (142)	105 hips (102)
Sex, male/female	26/116	19/83
Age (years)^a^	68.9 ± 9.8 (50–92)	69.2 ± 9.7 (50–92)
Height (cm)^a^	153.0 ± 7.7 (131–180)	153.0 ± 8.1 (131–180)
Weight (kg)^a^	56.3 ± 11.6 (33–102)	55.3 ± 11.1 (33–84)
Body mass index (kg/m^2^)^a^	23.9 ± 4.3 (14.7–40.2)	23.5 ± 4.1 (14.7–38.3)
Preoperative diagnosis		
Osteoarthritis (OA)	108	79
Osteonecrosis of the femoral head	15	8
Rheumatoid arthritis	9	6
Femoral neck fracture	17	12
The severity of knee OA		
grade 1	13	8
grade 2	56	39
grade 3	60	43
grade 4	20	15

^a^Mean ± SD (range)

Between December 2017 and November 2018, cemented stems were implanted using the angle-measuring instrument during THA. *Implantation accuracy* was defined as the difference between the target stem anteversion angle and the postoperative measurements of stem anteversion obtained using postoperative CT. Each cemented stem was placed at the target angle using the angle-measuring instrument, and the implantation accuracy of the angle-measuring instrument was investigated in 105 consecutive hips (Table 1).

### Surgical technique and measurements

**Fig. 1:**
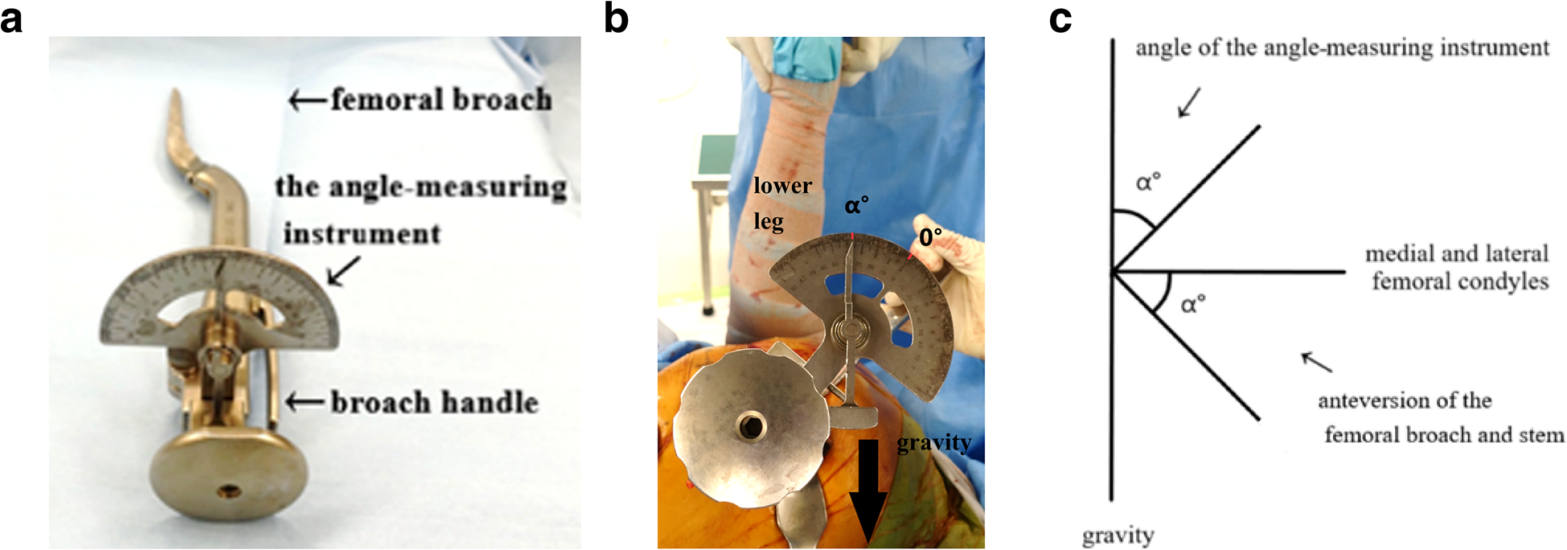
**a** The angle-measuring instrument attached to the handle of the femoral broach. **b** The angle of the angle-measuring instrument. The angle of the angle-measuring instrument was measured as the angle between the gravitational direction and the line running perpendicular to the prosthetic axis under 90° hip flexion, maximum hip adduction, and 90° knee flexion, with the tibia in a vertical position. α° represents the anteversion of the femoral broach and stem. **c** Anteversion of the femoral broach and stem. When the tibia was placed in a vertical position, the axis passing through the medial and lateral femoral condyles ran perpendicular to the gravitational direction. The anteversion of the femoral broach and stem was determined as the angle between the prosthetic axis and the line running perpendicular to the gravitational direction. The anteversion of the femoral broach and stem reflected the angle between the gravitational direction and the line running perpendicular to the prosthetic axis. α° indicates the anteversion of the femoral broach and stem

A standard posterolateral approach was used in the lateral decubitus position in all cases. A cementless cup (Continuum Cup; Zimmer, Warsaw, IN) and a cemented stem (CMK Original Concept Stem; Zimmer, Warsaw, IN) were used in all cases. The angle-measuring instrument was attached to the handle of the femoral broach and stem, which resulted in it being aligned perpendicularly to the femoral broach and stem (Fig. 1a). The pointer of the angle meter turns upward due to gravity. The anteversion of the femoral broach and stem was measured as the angle between the gravitational direction and the line running perpendicular to the prosthetic axis (the femoral broach or stem) under 90° hip flexion, maximum hip adduction, and 90° knee flexion, with the tibia placed in the vertical position (Fig. 1b and c).

The acetabular side was prepared first. Our target cup position for all patients was 40° abduction and 20° anteversion, similar to Domb et al. [19]. And then femoral preparation was performed. Intraoperatively femoral canal was reamed and broached. All final femoral rasps used in this study for canal preparation over-rasped by 0.8 mm [20]. Cement was packed into the femoral canal with a cement gun, and the stem was inserted. Care was taken to insert the stem in a central position. After the stem implantation, stem anteversion was measured and measurement accuracy was investigated. The intraoperative measurements of stem anteversion were compared with postoperative measurements of stem anteversion obtained using postoperative CT to investigate measurement accuracy. All of the intraoperative measurements were performed by the same assistant surgeon.

When the final femoral broach was placed, the angle-measuring instrument was used to measure the anteversion of the femoral broach, and the target angle was decided. A recent study reported that the intraoperative stability test, especially measuring the IR angle (the range of internal rotation under 90° hip flexion and 0° abduction/adduction), is a useful method for predicting hip stability after THA. An IR angle of 51° was set as the cut-off point for such predictions [21]. After removing the acetabular osteophytes and femoral neck remnants, the target angle was decided by fine-tuning the femoral broach and neck length to acquire an IR angle of > 51° without causing anterior instability. The cemented stem was placed at the target angle using the angle-measuring instrument, and the implantation accuracy of this method was investigated.

All patients underwent similar closure procedures and postoperative care. Postoperatively, stem anteversion was measured on CT. CT were obtained on all patients 1 week after surgery. Using a Multi slice CT scanner Aquilion 64 (TSX-101A) (Canon Medical Systems Co, Japan), consecutive scans, at 2-mm intervals, were performed from the level of the fourth lumbar vertebra proximally to the knee, including the entire distal femoral condyles. Stem anteversion was determined as the angle between the stem neck axis and the axis passing through the medial and lateral femoral condyles [22, 23]. All CT-based measurements of stem anteversion were performed by the same observer, and were repeated in a blind manner during the course of two sessions, which were at least 1 month apart. Intraobserver reliability was evaluated using the intraclass correlation coefficient. In addition, two observers independently made CT-based measurements, and interobserver reliability was evaluated using the interclass correlation coefficient.

The factors studied included sex, age, body mass index (BMI), the preoperative diagnosis, and the severity of knee osteoarthritis (OA). We divided the preoperative diagnoses into two groups, the OA group and non-OA group (osteonecrosis of the femoral head, rheumatoid arthritis, or femoral neck fractures) (Table 1). Knee OA was graded according to the classification of Kellgren and Lawrence [24] and then was divided into two stages, the early stage (grade 1 or 2) and the advanced stage (grade 3 or 4) (Table 1).

### Statistical analyses

The data are reported using descriptive statistics, including mean, standard deviation, and range values. The normality of the data was assessed using the Shapiro-Wilk test, and the paired t-test, non-parametric Mann-Whitney U test, and correlation analyses were performed to evaluate measurement accuracy and implantation accuracy. Univariate and multivariate linear regression analyses were conducted to analyze the potential risk factors that might affect the measurement error in measurement accuracy and implantation accuracy. *P*-values of < 0.05 were considered significant. All statistical analyses were performed using SPSS version 25 (SPSS Inc., Chicago, IL).

## Results

**Fig. 2:**
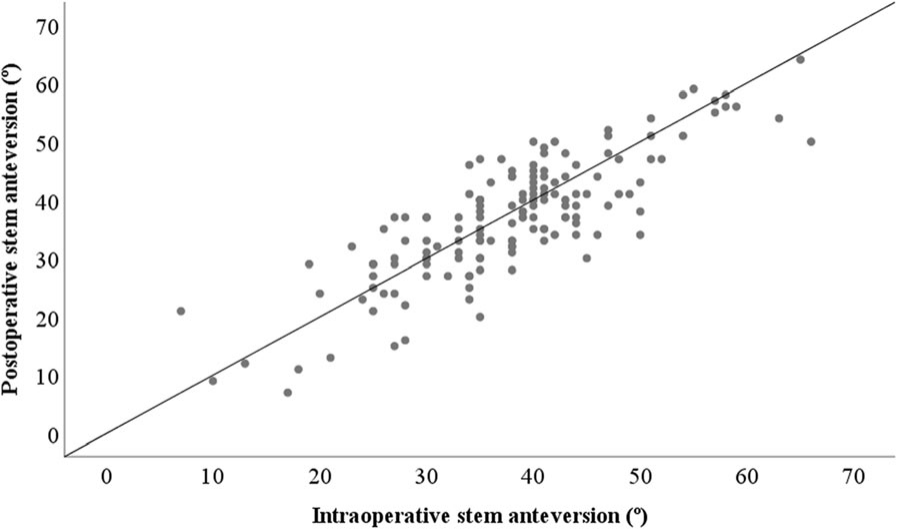
The correlation between intraoperative and postoperative stem anteversion measurements. The intraoperative stem anteversion measurements exhibited a good correlation with the postoperative stem anteversion measurements (correlation coefficient: γ = 0.824, *p* = 0.000)

The mean amount of intraoperative stem anteversion, as measured using the angle-measuring instrument, was 37.9° ± 10.1° (range: 7°–66°), and the mean amount of postoperative stem anteversion, as measured on CT, was 37.0° ± 10.4° (range: 7°–64°). The mean measurement accuracy (intraoperative stem anteversion - postoperative stem anteversion) was 0.9° ± 6.1° (range: − 14°–16°). The mean absolute measurement accuracy (|intraoperative stem anteversion - postoperative stem anteversion|) was 4.9° ± 3.7° (range: 0°–16°). The measurement accuracy was within 5° in 93 hips (62%) and within 10° in 138 hips (93%). The correlation coefficient for the relationship between the intraoperative and postoperative stem anteversion was 0.824 (*p* = 0.000) (Fig. 2). Among the factors analyzed in the univariate analyses, the severity of knee OA was found to be significantly related to measurement accuracy (p = 0.000). Sex, age, BMI, and the preoperative diagnosis were not found to be significantly related to measurement accuracy (*p* = 0.865, 0.267, 0.089, and 0.553, respectively). Multivariate analysis showed that the severity of knee OA significantly influenced measurement accuracy (Table 2). The mean measurement accuracy in the early stage knee OA group was 0.6° ± 4.5° (range: − 12°–16°), whereas it was 1.2° ± 7.2° (range: − 14°–16°) in the advanced stage knee OA group (*p* = 0.550). The mean absolute measurement accuracy in the early stage knee OA group was 3.3° ± 3.2° (range: 0°–16°), whereas it was 6.2° ± 3.5° (range: 0°–16°) in the advanced stage knee OA group (*p* = 0.000).

**Table 2:** Multivariate linear regression analysis to identify factors that affect measurement accuracy

Parameters	B	SE	β	t	*P*-value
Sex	0.135	0.792	0.034	0.452	0.652
Age	−0.034	0.031	−0.009	−0.118	0.906
BMI	−0.118	0.069	−0.119	−1.574	0.118
Preoperative diagnosis	0.406	0.683	0.026	0.338	0.736
Severity of knee OA	2.883	0.564	0.388	5.111	0.000^*^

B: unstandardized coefficient; SE: standard error; β: standardized coefficient; ^*^p < 0.05

**Fig. 3:**
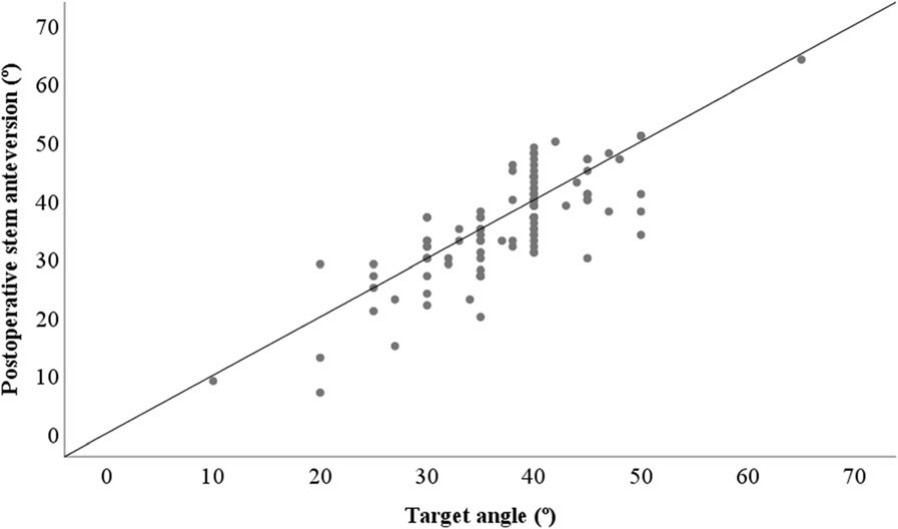
The correlation between the target angle and the postoperative stem anteversion measurements. The target angle exhibited a good correlation with the postoperative stem anteversion measurements (correlation coefficient: γ = 0.795, *p* = 0.000)

The mean amount of target angle was 37.4° ± 7.6° (range: 10°–65°), and the mean amount of postoperative stem anteversion, as measured on CT, was 35.9° ± 9.1° (range: 7°–64°). The mean implantation accuracy (target angle - postoperative stem anteversion) was 1.4° ± 5.6° (range: − 9°–16°). The mean absolute implantation accuracy (|target anteversion - postoperative stem anteversion|) was 4.3° ± 3.6° (range: 0°–16°). The implantation accuracy was within 5° in 71 hips (68%) and within 10° in 98 hips (93%). The correlation coefficient for the relationship between the target angle and postoperative stem anteversion was 0.795 (*p* = 0.000) (Fig. 3). Among the factors analyzed in the univariate analyses, age and the severity of knee OA were found to be significantly associated with implantation accuracy (*p* = 0.049 and 0.000, respectively). Sex, BMI, and the preoperative diagnosis were not found to be significantly related to implantation accuracy (*p* = 0.850, 0.066, and 0.267, respectively). Multivariate analysis showed that the severity of knee OA significantly influenced implantation accuracy (Table 3). The mean implantation accuracy in the early stage knee OA group was 0.7° ± 3.4° (range: − 8°–9°), whereas it was 2.2° ± 6.7° (range: − 9°–16°) in the advanced stage knee OA group (*p* = 0.157). The mean absolute implantation accuracy was 2.7° ± 2.6° (range: 0°–9°) in the early stage knee OA group, whereas it was 5.8° ± 4.0° (range: 0°–16°) in the advanced stage knee OA group (*p* = 0.000).

**Table 3:** Multivariate linear regression analysis to identify factors that affect implantation accuracy

Parameters	B	SE	β	t	p-value
Sex	0.177	0.934	0.050	0.558	0.578
Age	0.073	0.036	0.100	1.089	0.279
BMI	−0.160	0.086	−0.121	−1.348	0.181
Preoperative diagnosis	0.925	0.828	0.079	0.884	0.087
Severity of knee OA	3.078	0.657	0.419	4.687	0.000^*^

B: unstandardized coefficient; SE: standard error; β: standardized coefficient; ^*^p < 0.05

Excellent intraobserver reliability was seen; i.e., the intraclass correlation coefficient was 0.983 (range: 0.977–0.988). In addition, excellent interobserver reliability was observed; i.e., the interclass correlation coefficient was 0.948 (range: 0.847–0.981).

No intraoperative or postoperative complications related to the measurements were noted.

## Discussion

We investigated the measurement accuracy of the angle-measuring instrument for measuring the angles of cemented stems during THA and examined the accuracy of stem positioning performed using the angle-measuring instrument. The mean absolute measurement accuracy was 4.9°, and the mean absolute implantation accuracy was 4.3°. As for implantation accuracy, 68% of cases were within 5°, and 93% were within 10°.

Generally, the intraoperative estimation of stem anteversion is performed based on surgeons’ visual assessments. Several studies have reported that there can be considerable differences between surgeons’ visual assessments of stem anteversion and postoperative measurements of stem anteversion [18, 22, 25]. Wines and McNichol reported that the mean difference between surgeons’ visual assessments of stem anteversion and postoperative measurements of stem anteversion was an underestimation of 1.1°, but the standard deviation was 10.4° and a range of 25° underestimation to 30° overestimation. A high standard deviation means that the data were widely spread (less reliable). They believed that these differences were clinically unacceptable [22]. Dorr et al. reported that there was a weak correlation between surgeons’ visual assessments of stem anteversion and postoperative measurements of stem anteversion [18]. In addition, Woerner et al. reported that the mean difference between surgeons’ visual assessments of stem anteversion and postoperative measurements of stem anteversion was an underestimation of 7.3° ± 9.8° [25].

Several studies have investigated the accuracy of measurements obtained using a goniometer or navigation systems during THA [12–16] (Table 4). They reported that the mean absolute measurement accuracy ranged from 4.5°–7.3°. Thus, the mean absolute measurement accuracy value obtained in the present study (4.9°) seems to be acceptable. Several studies have reported that knee OA and the femorotibial angle significantly influenced measurement accuracy during THA [13, 16]. In the current study, knee OA was shown to significantly influence measurement accuracy during THA, which is consistent with the other studies.

**Table 4:** Measurement accuracies reported in the literature

Studies	Type	Intraoperative stem anteversion	Postoperative stem anteversion	Mean absolute measurement accuracy	Correlation coefficient
Hirata et al. [13]	goniometer	40.5 ± 11.6°	34.7 ± 11.2°	7.3 ± 5.7°	0.798
Fujihara et al. [15]	goniometer	22.7 ± 8.4°	19.9 ± 8.4°	4.6 ± 4.1°	
Lee et al. [16]	goniometer	21.5 ± 8.5°	19.5 ± 8.7°	4.5 ± 2.8°	0.837
Hirasawa et al. [12]	CT-based navigation			5.2 ± 4.8°	
Fukunishi et al. [14]	Imageless navigation	15.5 ± 9.7°	20.9 ± 10.6°	5.9 ± 4.2°	
Current study	Angle-measuring instrument	37.9 ± 10.1°	37.0 ± 10.4°	4.9 ± 3.7°	0.824

Only one CT-based navigation study has evaluated stem implantation accuracy during THA [17], and we could not find any clinical studies that examined stem implantation accuracy using a goniometer or mechanical guides. Kitada et al. reported that the mean target angle was 34.2° ± 12.4°, and the mean postoperative stem anteversion was 31.7° ± 11.7°. Thus, the mean implantation accuracy value was − 2.5° ± 6.1°. The absolute implantation accuracy was not reported. Our mean implantation accuracy value was 1.4° ± 5.6°, which is similar to the abovementioned value. In a previous navigation study, it was reported that stem anteversion implantation accuracy of within 5° was achieved in 60% of cases. In our study, stem anteversion implantation accuracy of within 5° was achieved in 68% of cases, suggesting that the angle-measuring instrument can be used to achieve accurate stem implantation. Typically, the surgical navigation systems need a large computer console, need optical sensors or magnetic sensors to track the bones and surgical tools or implant, and are expensive. Our angle-measuring instrument is simple, does not need a large computer console and sensors, is easy to use, and requires no metal fitting entry into the body compared with navigation systems. However, the stem anteversion implantation accuracy was > 5° in almost 30% of cases. This suggests that the angle-measuring instrument should be used with caution, and that the instrument requires further improvement.

Improvements in our understanding of femoral morphology and anteversion and their influence on implant impingement and dislocation have caused surgeons to re-evaluate component positioning during THA [26]. Several studies have examined stem anteversion during THA [18, 27]. Dorr et al. proposed that stem anteversion should exhibit an approximate range of 10°–20°, whereas D’Lima et al. stated that it should display an approximate range of 10°–30°. The mean target angle in the present study was 37.4°. Although this was larger than those described in other studies, it was similar to some previously target angle [17]. As the present study included many cases of developmental dysplasia of the hip, in which the degree of femoral anteversion was significantly large, we employed larger target angles [28]. Recently, the concept of the IR angle was reported to be a useful method for predicting hip stability after THA, and an IR angle of 51° was suggested to be an appropriate cut-off point for such predictions [21]. Because a recent study reported that stem anteversion had the strongest effect on the IR angle [29], we fine-tuned stem anteversion prior to the final implantation to achieve an appropriate IR angle. Therefore, it is necessary to obtain precise intraoperative information about stem anteversion, and it is very important that surgeons are able to place cemented stems accurately during THA.

Our study had several limitations. First, it did not involve a case-control group. Second, only the posterolateral approach was examined. Danoff et al. reported that when a posterior approach is employed the cup anteversion safe zone is larger than was previously believed [30]. Thus, the optimal degree of stem anteversion might differ according to the approach employed. Third, the target angle was decided by fine-tuning the femoral broach to acquire an appropriate IR angle; however, the concept of combined anteversion has been proposed [31, 32]. Fourth, we checked the vertical position of the tibia in visual assessment during the intraoperative measurement of stem anteversion. Although this was similar to other clinical studies [13, 16, 18, 25], it can affect the accuracy of measurements. Finally, we did not evaluate postoperative clinical outcomes; however, this was beyond the purpose of this study.

## Conclusions

We developed a simple angle-measuring instrument to measure cemented stem anteversion intraoperatively and to place cemented stems accurately during THA. The measurement accuracy was 4.9°, that was similar to other studies with goniometer and navigation systems. And the implantation accuracy was 4.3°, that was similar to one navigation study. However, the stem anteversion implantation accuracy was > 5° in almost 30% of cases, and the instrument requires further improvement.

## Data Availability

The datasets used and/or analysed in this study are available from the corresponding author on reasonable request.

## Abbreviations

BMI: Body mass index
CT: Computed tomography
OA: Osteoarthritis
THA: Total hip arthroplasty
